# Climate Change and Infectious Disease

**DOI:** 10.1155/2009/976403

**Published:** 2009-04-15

**Authors:** Bettina C. Fries, Jonathan Mayer

**Affiliations:** ^1^Albert-Einstein College of Medicine Bronx, Yeshiva University, New York, NY 10461, USA; ^2^University of Washington, Seattle, WA 98195, USA

Due to increasing CO_2_ emissions and other greenhouse gases, accelerated global warming is predicted by the Intergovernmental Panel on Climatic Change (IPCC). These climate changes are anticipated to have a long-term impact on marine and terrestrial ecosystems. Rising sea levels are predicted to flood low-lying coastal regions, and fresh water resources could become scarce leading to development of desert-like regions. This will greatly affect the survival of fragile plant species, wild animals, and other ecosystems all of which could directly or indirectly affect survival and or spread of pathogens and their vectors. Although these changes will undoubtedly have an impact on human health, the uncertainties in climate models, as well as predictive epidemiology, hamper accurate predictions on the impact of global warming on public health. In addition, the absence of experimental models that demonstrate dose dependent effects between climate change and disease gives ammunition to those in doubt. As a joint Institute of Medicine/National Academy of Science Committee concluded in 2001, we know that there is a relationship between climate change and infectious disease, but these links are disease specific and location specific, and we cannot yet predict these impacts at anything other than coarse temporal and spatial scales. Thus, the impact on human health is still unclear, and climate change may increase the prevalence of particular infectious diseases in some regions, while decreasing the prevalence in others. There are currently 137 publications in PubMed using the keywords “infectious diseases” and “global warming.” The majority of which were published in the last few years, and this attests to the increasing interest in this aspect of public health. However, most data so far are observational, and the predictive understanding of the impact of disease is based on mathematical modeling. Other sources of data include retrospective time-series analyses in specific locations, or use the periodic El Nino Southern Oscillation (ENSO) phenomenon as a surrogate for long-term change. Since controls are usually historical and data are now collected in a more biased fashion than before, there is a concern that some of our conclusions reflect an observational bias that is common when data collection is initiated on new problems that were not previously under rigid observation.

To tackle global warming, coordinated efforts will have to be taken that do not only span countries and cultures but also require many fields of scientific expertise. Effects of global warming are multifactorial and occur at a time when many other important confounding variables constantly change. Beyond global warming, increasing global mobility, genetic modification of agricultural products, and usage of reagents that inhibit pathogens can all have a significant impact on spread and prevalence of infectious diseases. This will make it difficult to establish causality between global warming and impact of infectious diseases. Rigorous scientific conclusions call for experimental models that examine impact of temperature under controlled conditions. Finally, global warming occurs in the general setting of climate change—temperature is not the only variable that is changing. Rather, rainfall patterns, wind patterns, and other climatologic variables are changing at the same time.

As much as we all wish to act fast, and as much as we cry out for an enlightened public consciousness that would be required to press for policy changes that ameliorate the effects of global warming and limit the emission of greenhouse gases, our most imminent problem is that we do not have a true scientific understanding of the matter yet. In this special issue, Interdisciplinary Perspectives on Infectious Diseases solicited cross-cutting interdisciplinary articles that took new and broad perspectives ranging from what we might learn from previous climate changes on disease spread to integrating evolutionary and ecologic theory with epidemiologic evidence in order to identify key areas for study in order to predict the impact of ongoing climate. These studies present interesting results and analysis of available data. They also highlight how difficult it is to interpret data at present.

Future studies should increase retrospective surveys of museum specimens from previous times, long lasting unbiased data mining and true experimental work on disease manifestation under higher temperatures. Moreover, predictive climate models and predictive epidemiology both need to advance, and become coupled together. This research will likely not meet current output criteria of short-term grant proposals. This problem needs meticulous patient intelligent researches like Darwin to devote their life to answering these pressing questions. Without such efforts, the current generation will inevitably benefit at the cost of generations to come.

*Bettina C. Fries* *Bettina C. Fries* *Jonathan Mayer* *Jonathan Mayer*

